# Effect of colistin-tigecycline combination on colistin-resistant and carbapenem-resistant *Klebsiella pneumoniae* and *Acinetobacter baumannii*

**DOI:** 10.1128/spectrum.02021-24

**Published:** 2024-12-19

**Authors:** Suyeon Park, Yanhong Jin, Kwan Soo Ko

**Affiliations:** 1 Department of Microbiology, Sungkyunkwan University School of Medicine, Suwon, South Korea; The University of Sydney, Darlington, New South Wales, Australia

**Keywords:** colistin, carbapenem resistance, tigecycline, *Klebsiella pneumoniae*, *Acinetobacter baumannii*

## Abstract

There is a critical need for treatment strategies to combat carbapenem-resistant Gram-negative pathogens. This study investigates the efficacy of combining low concentrations of colistin with tigecycline against colistin- and carbapenem-resistant *Klebsiella pneumoniae* and *Acinetobacter baumannii* strains. We used two strains of KPC-2-producing *K. pneumoniae* and OXA-23-producing *A. baumannii*, both of which are highly colistin-resistant. In the *in vitro* time-killing assays, monotherapy with colistin (2 mg/L) and tigecycline (4 or 8 mg/L) was ineffective against all strains. However, the combination of colistin (2 mg/L) and tigecycline (4 or 8 mg/L) inhibited bacterial growth. Survival analysis of *Galleria mellonella* larvae also demonstrated the efficacy of low concentrations of colistin and tigecycline against colistin- and carbapenem-resistant strains. All larvae survived when treated with a combination of colistin and tigecycline, whereas monotherapy with either colistin or tigecycline did not increase larval survival rates. This study proposes using a combination of tigecycline and colistin at clinical concentrations to treat carbapenem-resistant *K. pneumoniae* and *A. baumannii,* regardless of colistin resistance.

**IMPORTANCE:**

Colistin resistance in carbapenem-resistant Gram-negative pathogens is a serious challenge in clinical settings. Our study showed that low concentration of colistin can be effective when combined with tigecycline. It presents the possibility of an available treatment option for antibiotic-resistant pathogens.

## INTRODUCTION

*Klebsiella pneumoniae* and *Acinetobacter baumannii* are notorious nosocomial pathogens that frequently cause infections in hospitals and long-term care facilities (1). These pathogenic infections can lead to severe outcomes, including pneumonia, bloodstream infections, wound or surgical site infections, and meningitis (2, 3). Particularly concerning are carbapenem-resistant *K. pneumoniae* (CRKP) and carbapenem-resistant *A. baumannii* (CRAB), which pose significant threats to global public health owing to their high levels of resistance to multiple antibiotics, severely limiting treatment options (4).

Carbapenem resistance in these pathogens is primarily mediated by the production of carbapenemases, which hydrolyze the antibiotics, rendering them ineffective (5). This resistance mechanism, coupled with the ability of bacteria to acquire additional resistance genes, poses a serious challenge in clinical settings, leading to increased morbidity, mortality, and healthcare costs (6).

Considering the urgency of this situation, there is a critical need for novel treatment strategies to combat CRKP and CRAB infections. One promising approach involves the use of colistin, a polymyxin antibiotic often considered a last-resort treatment for multidrug-resistant Gram-negative infections (7). However, the emergence of colistin resistance complicates the treatment landscape. Previous studies have observed that exposure to tigecycline could reverse colistin resistance in *K. pneumoniae* (8). This intriguing finding prompted further investigations into whether this phenomenon could be applied to the treatment of CRKP and CRAB infections. In this study, we investigated the efficacy of low-dose colistin in combination with tigecycline for the treatment of CRKP and CRAB infections.

## RESULTS

All CRKP and CRAB strains showed susceptibility to tigecycline but resistance to colistin, meropenem, and imipenem (Table 1). Both CRKP strains produced KPC-2 and both CRAB strains produced OXA-23. All the strains tested negative for the *mcr* gene. Their colistin resistance was identified to be due to the increased expression of *pmrAB*, leading to L-Ara4N.

**TABLE 1:** Antimicrobial susceptibility profiles of *K. pneumoniae* and *A. baumannii* strains

Species	Strain	Minimum inhibitory concentration (mg/L)^*a*^			
		Tigecycline	Colistin	Meropenem	Imipenem
*K. pneumoniae*	742	1 (S)	64 (R)	64 (R)	64 (R)
	777	1 (S)	64 (R)	64 (R)	64 (R)
*A. baumannii*	F-1629	2 (S)	>64 (R)	>64 (R)	>64 (R)
	SCH2203-16	2 (S)	64 (R)	>64 (R)	>64 (R)

^*a*^ R, resistant; S, susceptible.

**Fig 1:**
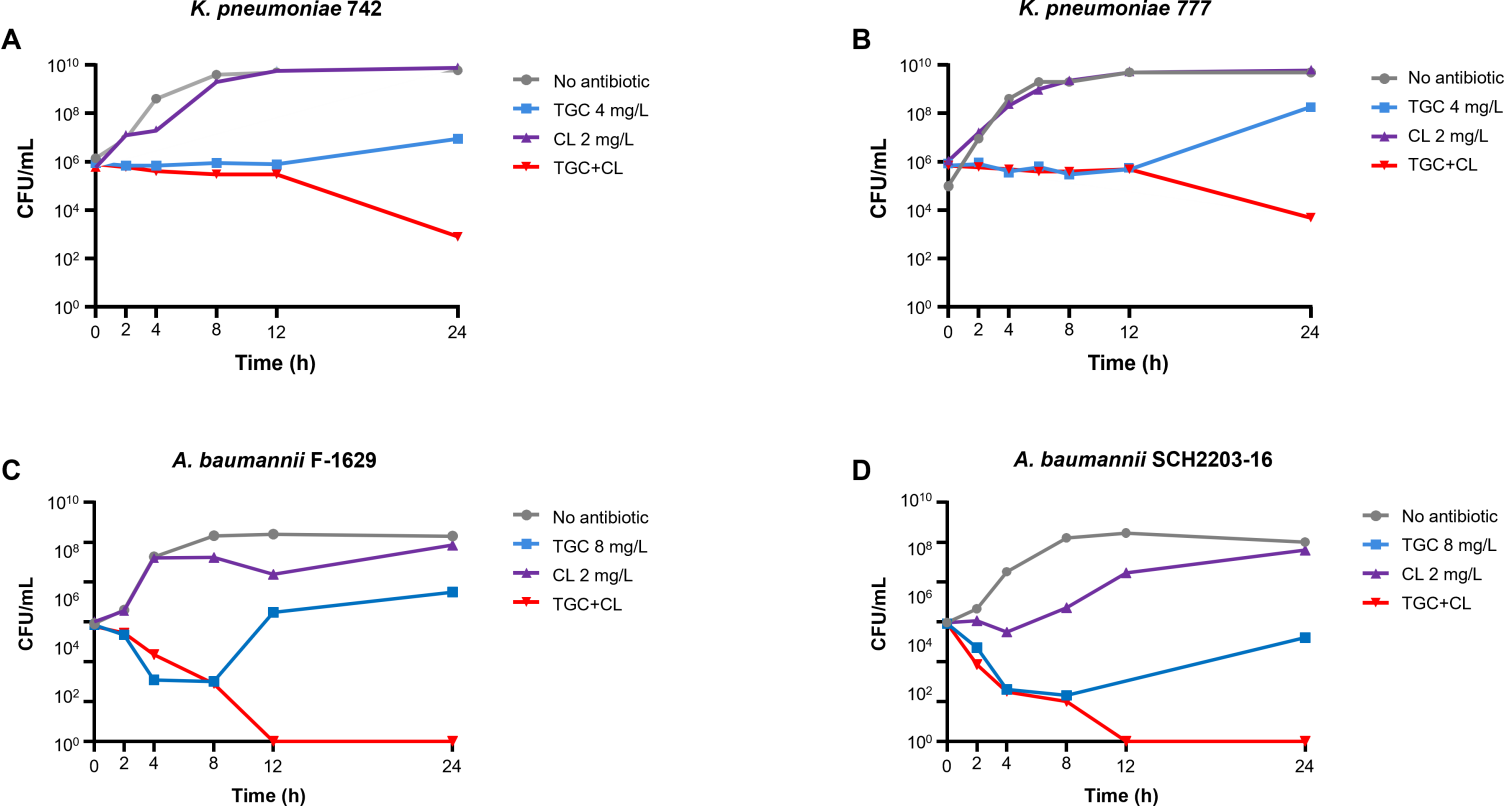
The results of *in vitro* time-killing assay. For all strains, colistin was administered at 2 mg/L, while tigecycline was administered at 4 mg/L (*K. pneumoniae*) or 8 mg/L (*A. baumannii*), corresponding to 4× MIC. (A) *K. pneumonmiae* 742; (B) *K. pneumoniae* 777; (C) *A. baumannii* F-1629; (D) *A. baumannii* SCH2203-16.

Time-killing assays were performed on all strains using single regimens (colistin or tigecycline) or a combination of colistin and tigecycline. Colistin was equally administered at 2 mg/L despite colistin resistance, and tigecycline was administered at 4 or 8 mg/L, which corresponded to 4× MIC. As expected, a low concentration of colistin did not inhibit the growth of colistin-resistant CRKP and CRAB strains, showing no obvious changes compared to those without antibiotic treatment (Fig. 1). For tigecycline, no growth inhibition was observed in either CRKP strains (Fig. 1A and B). The growth of the CRAB strains was inhibited upon exposure to tigecycline; however, regrowth occurred after 8 h of treatment (Fig. 1C and D).

Unlike the single regimens, the combination of low concentrations of colistin and tigecycline inhibited bacterial growth despite colistin resistance (MICs, 64 or >64 mg/L). In particular, the colistin-resistant CRAB strains were completely eradicated within 12 h of treatment (Fig. 1C and D). The growth of the colistin-resistant CRKP strains was also greatly reduced compared to those with no treatment or treatment with a single regimen (Fig. 1A and B).

**Fig 2:**
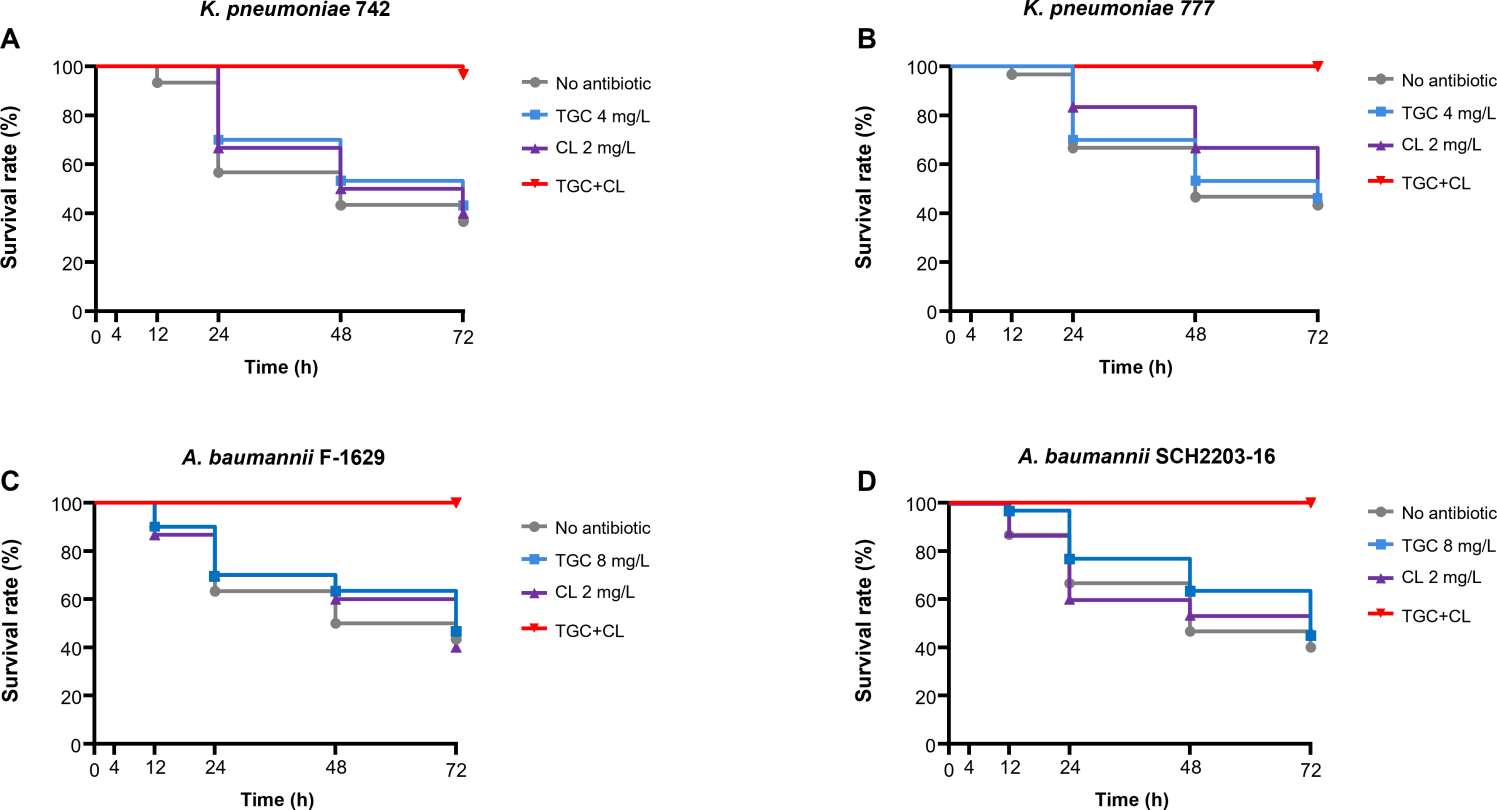
The results of survival analysis of *G. mellonella* larvae. The larvae were infected with *K. pneumoniae* at 1 × 10^6^ colony-forming unit and *A. baumannii* at 1 × 10^7^, corresponding to their LD_50_. They were treated with colistin at 2 mg/L and tigecycline at 4 mg/L (*K. pneumoniae*) or 8 mg/L (*A. baumannii*), which correspond to 4× MIC. (A) *K. pneumonmiae* 742; (B) *K. pneumoniae* 777; (C) *A. baumannii* F-1629; (D) *A. baumannii* SCH2203-16.

Experiments with *G. mellonella* larvae demonstrated the efficacy of low concentrations of colistin and tigecycline against colistin-resistant CRKP and CRAB strains (Fig. 2). *G. mellonella* larvae were infected with the LD_50_ of CRKP and CRAB strains. They were then administered single regimens and combinations as in *in vitro* time-killing assays, and larval survival rates were monitored for 72 h. Without antibiotic treatment, only 40%–50% of the infected *G. mellonella* larvae survived. Monotherapy with colistin (2 mg/L) or tigecycline (4 or 8 mg/L) showed no difference in therapeutic efficacy. However, all larvae survived when administered combinations of low concentrations of colistin and tigecycline, despite the bacterial strains’ high colistin resistance.

## DISCUSSION

Carbapenem resistance in *K. pneumoniae* and *A. baumannii* severely limits therapeutic options, primarily relying on restricted antibiotics such as colistin. Consequently, colistin resistance in CRKP and CRAB cells poses a significant challenge for treatment. While tigecycline is also used to treat CRKP and CRAB infections, its efficacy in monotherapy is often questioned due to low serum concentrations and limited penetration in the epithelial lining fluid (9). Therefore, combination therapies with other antibiotics are strongly recommended. Particularly, the tigecycline-colistin combination has demonstrated efficacy both *in vitro* and *in vivo* against CRKP and CRAB (10, 11). However, colistin-based combination therapy has not been actively proposed for colistin-resistant pathogens because it is assumed to be ineffective.

In this study, we showed that a tigecycline-colistin combination could effectively treat colistin-resistant CRKP and CRAB strains. Despite MICs of colistin being 64 mg/L or higher in the CRKP and CRAB strains, the combination of 2 mg/L colistin combined with tigecycline exhibited an excellent *in vitro* killing effect. *In vivo* survival analysis using *G. mellonella* larvae further showed that all larvae infected with bacteria survived when treated with low concentrations of colistin and tigecycline.

The reason for the effective killing effect of low concentrations of colistin combined with tigecycline, despite colistin resistance, remains unclear. While colistin is bactericidal, tigecycline acts as bacteriostatic. Tigecycline inhibits protein synthesis in bacteria, leading to alterations in the bacterial cells that they encounter. Subsequently, colistin may induce bacterial cell death by disrupting the cell membrane. It is hypothesized that the synergistic effect between these two antibiotics occurs within a timeframe of 8–12 h. Colistin has been shown to amplify the effects of other antibiotics by partially cracking the cell walls and membranes (12, 13). Although the concentration (2 mg/L) of colistin could not kill colistin-resistant bacterial strains, it may enhance bacterial cell death by facilitating tigecycline penetration into the cell.

In summary, we demonstrated the possibility of combining low concentrations of colistin with tigecycline to treat colistin-resistant CRKP and CRAB strains.

## MATERIALS AND METHODS

### Bacterial strains and antibiotic susceptibility testing

Two CRKP strains, 742 and 777, and two CRAB strains, F-1629 and SCH2203, were used. These strains were isolated from the blood of patients at Daegu Fatima Hospital (Daegu, South Korea) and Samsung Changwon Hospital (Changwon, South Korea).

Antimicrobial susceptibility testing for tigecycline, colistin, meropenem, and imipenem was performed using the broth microdilution method in accordance with the Clinical Laboratory and Standards Institute (14). *Escherichia coli* ATCC 25922 and *Pseudomonas aeruginosa* ATCC 27853 were used as controls. All tests were conducted in duplicate.

### *In vitro* time-killing assays

*In vitro* time-killing assays were performed to monitor the combined efficiency of tigecycline and colistin at low concentrations against CRKP and CRAB strains. Time-kill tests were conducted using Luria-Bertani (LB) broth as the control growth medium. All strains were grown overnight at 37°C with shaking. Bacterial cultures were then adjusted to a McFarland 0.5 standard with PBS before being subcultured in LB broth containing 4× MIC of tigecycline, 2 mg/L of colistin, or a combination of both. Colony-forming units were quantified at 0, 2, 4, 8, 12, and 24 h by plating serial dilutions of the samples on LB plates. Each experiment was performed in duplicate and repeated three times independently.

### *Galleria mellonella* infection and treatment

To determine the efficacy of combination therapy with tigecycline and colistin at low concentrations, *Galleria mellonella* infection model was used, with minor modifications (15). *G. mellonella* larvae were purchased from Sworm Corp. (Cheonan, South Korea) and kept at room temperature in the dark with food for 5 days before infection. Bacterial infections of *G. mellonella* were performed with 10 larvae, using PBS injection as a positive control. Overnight bacterial cultures were harvested by centrifugation at 13,000 rpm for 2 min, subsequently washed with PBS, and adjusted to 1 × 10^6^ (LD_50_) for *K. pneumoniae* and 1 × 10^7^ (LD_50_) for *A. baumannii*. Ten larvae were infected with 10 µL of each bacterial solution by injection into the last right proleg using an ultra-fine needle (BD Biosciences, San Jose, CA, USA). After 30 min of bacterial infection, 4× MIC of tigecycline, 2 mg/L colistin, or a combination of both was injected into the infected larvae. The larvae were examined for up to 72 h post-infection. All tests were conducted three times independently.

### Statistical analysis

Statistical analysis was performed using Student’s *t*-test with Prism version 8.3 for Windows (GraphPad Software, San Diego, CA, USA). *P*-values of <0.05 were considered statistically significant (*P < 0.05; **P < 0.001; ***P < 0.0001; and ****P < 0.00001).
